# Design and evaluation of a magnetic resonance‐guided focused ultrasound system for the treatment of cervical spine pain

**DOI:** 10.1002/mp.70221

**Published:** 2025-12-21

**Authors:** Viola Rieke, Allison Payne, Robb Merrill, J. Rock Hadley, Henrik Odéen, Michelle Kline, Matthew S. Zabriskie, Lubdha M. Shah

**Affiliations:** ^1^ Department of Radiology and Imaging Sciences University of Utah Salt Lake City Utah USA; ^2^ Department of Biomedical Engineering University of Utah Salt Lake City Utah USA

**Keywords:** ablation, cervical spine, focused ultrasound, magnetic resonance imaging

## Abstract

**Background:**

Neck pain is a common health problem, with a large percentage of individuals experiencing chronic or recurring pain. A considerable portion of chronic neck pain is attributed to the facet joints, which are small joints located at the back of the spinal column. Current treatments for this pain range from conservative treatments to invasive surgeries, each of which has potential risks and limitations. While radiofrequency ablation can be effective, it remains invasive and carries the risk of radiation exposure and long‐term damage to paraspinal muscles. Magnetic resonance‐guided focused ultrasound (MRgFUS) offers a promising alternative by enabling non‐invasive and precise ablation of targeted tissue. However, the application of MRgFUS in the cervical spine requires careful consideration to avoid critical vascular and neural structures. Currently, there are no Food and Drug Administration approved focused ultrasound (FUS) devices for the cervical spine.

**Purpose:**

This study aimed to design and evaluate a neck‐specific MRgFUS system for the ablation of cervical facet joints in a preclinical setting. The goal was to demonstrate that MRgFUS could effectively target and ablate these joints while avoiding damage to surrounding critical structures.

**Methods:**

The study involved the development of a specialized MRgFUS system consisting of an adjustable positioning gantry, a head support frame that supports the subject's head and neck above the transducer assembly, a transducer adjustment, and acoustic coupling system. The system included integrated magnetic resonance (MR) radiofrequency (RF) receive coils to enhance signal‐to‐noise ratio (SNR) and ensure accurate imaging during the procedure. The system was tested on five goats, where the cervical facet joints were targeted for ablation. Transducer position was located with the positioning coils, and the ultrasound focus was determined with MR‐acoustic radiation force imaging (MR‐ARFI) in adjacent muscle. Ablation of the facet joint was monitored in real‐time with MR‐temperature imaging (MRTI).

**Results:**

The system demonstrated prediction accuracy of the geometric focus of 2.22 ± 0.74 mm. The MR imaging coil SNR was measured to be at least five times higher compared to the body coil. MR temperature precision was found to be 0.58 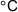. The system successfully targeted and ablated the cervical facet joints using MR‐ARFI and MRTI for guidance and monitoring.

**Conclusions:**

The neck‐specific MRgFUS system was able to target and ablate the cervical facet joints and monitor the treatment in real‐time. The results suggest that this system has the potential for non‐invasive treatment of facet joint related neck pain.

## 1 INTRODUCTION

Neck pain is the fourth leading cause of disability and nearly 50% of individuals with neck pain will experience chronic or recurring pain.^1^ Up to 67% of chronic neck pain involves the facet joints—small joints at the posterolateral aspect of the spinal column.^2^, ^3^, ^4^, ^5^, ^6^ Pain from facet joints often emanates to various parts of the neck, head, and upper shoulder, causing reduced quality of life and increased disability.^7^ Degeneration or trauma (often from whiplash) in the facet joints are the most common causes of cervical facet joint pathology and pain.^8^, ^9^, ^10^

Current treatments for cervical neck pain range from conservative treatments to surgical interventions. Conservative treatments include the administration of systemic analgesic drugs, such as opioids, and often fail due to their off‐target toxicity, capacity for tolerance development, and abuse potential.^11^, ^12^, ^13^, ^14^, ^15^ Highly invasive surgery aimed to correct anatomic abnormalities has inherent procedural and long‐term risks, including increased postsurgical pain.^16^, ^17^ Interventional pain procedures, such as radiofrequency ablation (RFA) of the facet joint, have higher specificity as they locally target the pain‐generating facet joints. RFA can result in pain relief lasting longer than 12 months in a majority of correctly selected patients. Despite its successes, RFA remains an invasive procedure with risk of radiation exposure from fluoroscopy guidance, infection or hematoma,^18^, ^19^, ^20^ and long‐term damage to paraspinal muscles.^21^, ^22^

The cervical facet joints (FJ) are innervated by medial branches of the dorsal rami from the same level and the level above and are RFA targets. Compared to FJ targets in the lumbar spine, however, neck percutaneous RFAs pose even greater risks of complications given the close proximity of critical vascular and neural structures to the osseous spine and the cervical spinal cord such that off‐target trajectory can be catastrophic. Major complications (stroke, carotid blowout) relating to the proximity of electrodes to the carotid artery have been reported with percutaneous RFA procedures.^23^ For cervicogenic headache, the targets for RFA are the third occipital nerve (TON) and an articular branch of the C3 posterior ramus, which innervate the C2‐3 FJ. These targets are precariously close to the vertebral arteries and neural structures (spinal cord). Procedures at these higher levels have adverse event rate of 18.5%.^24^

Image guidance can improve targeting for interventional pain procedures in the spine. Fluoroscopy is most often used in conjunction with RFA to identify osseous landmarks for facet RFAs. The inability to visualize the intervening structures increases the risk of complications due to imprecise targeting. Some studies have reported feasibility with computer tomography (CT) guidance,^25^ but radiation exposure to neck vascular structures, nerves, pharyngeal spaces, eyes, and brain can have long‐term negative effects. This is particularly concerning in treatment of cervicogenic headache. Magnetic resonance‐guidance (MRg) provides superior soft tissue contrast and resolution compared to fluoroscopy or CT guidance, which is advantageous in avoiding inadvertent vessel or nerve injury.

Focused ultrasound can be used in combination with magnetic resonance‐guidance (MRgFUS) and can ablate targeted tissue by inducing localized heating^26^ when applied at high intensity. There has been a rapid expansion of MRgFUS into a multitude of clinical applications including movement disorders,^27^, ^28^ uterine fibroids,^29^, ^30^, ^31^ painful bone metastases,^32^ breast cancer.^33^, ^34^ and desmoid tumors.^35^ MRgFUS provides the possibility of treating chronic neck pain by focally targeting the facet joints in a completely non‐invasive and low‐risk manner. Although MRgFUS holds promise for application in the neck, it has yet to be investigated if MRgFUS can focally ablate cervical facet joints while avoiding critical vascular and neural structures in the neck.

MRgFUS allows for non‐invasive and precise ablation of targets in the lumbar spine^36^, ^37^ and has been used as an effective and safe thermal ablation technique for outpatient management of FJ‐related low back pain.^38^, ^39^, ^40^ FUS performed under non‐ionizing MR‐guidance offers many benefits including better treatment visualization, excellent soft tissue characterization, three‐dimensional, and real‐time temperature imaging. These factors allow for accurate and precise targeting and treatment adjustments based on ongoing monitoring of the therapeutic effect on the tissue that assures maximum effectiveness and safety.

Currently, there are no Food and Drug Administration approved FUS devices for the spine. The Exablate system (in‐table transducer designed for uterine fibroid ablation; Insightec, Tirat–Carmel, Israel) has been used for facet joint ablation in the lumbar spine,^38^, ^39^, ^40^ but is not suited for bilateral treatments in the cervical spine due to limited transducer tilt angles and fixed location in the middle of the MR scanner table. Besides this MR‐guided system, an x‐ray guided system, Neurolyser XR (FUSMobile, Alpharetta, GA), is being developed for axial low back pain.^41^ This system is more flexible in transducer positioning for the lumbar spine, but may suffer from the same disadvantages as RFA in terms of safely targeting the cervical spine with x‐ray image guidance.

Previously, we have demonstrated in simulations that ablation of the cervical facet joints with MRgFUS transducers is safe and feasible.^42^ In this work, the design and preclinical evaluation of a neck‐specific MRgFUS device with integrated MR imaging coils that allows for bilateral treatment of the facet joints in the cervical spine is presented. The system is demonstrated in an in vivo goat model.

## 2 METHODS

The cervical spine MRgFUS system presented in this work was designed to demonstrate in a preclinical model that MRgFUS could thermally ablate three to four cervical facet joint levels (C2/3 through C6/7). Goats were chosen as the model because their neck diameter, vertebral body size and shape, and surrounding tissue are similar to human anatomy.^43^ The following sections describe the system components, bench‐top testing, and in vivo experiments. Although the system is designed for bilateral treatment, all in vivo experiments described in this work were done unilaterally with the collateral side undergoing RFA for comparison, which is not described in this manuscript.

### 2.1 Cervical spine MRgFUS system

**FIGURE 1:**
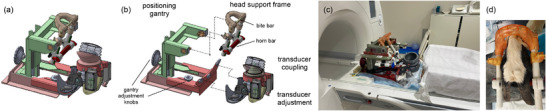
Cervical spine MRgFUS system: (a) Oblique complete schematic and (b) assembly views showing the positioning gantry, head support frame, and transducer adjustment and coupling system. (c) The installed system (transducer without coupling system) in a wide bore 3T scanner shows how the system is designed to integrate into the existing MR exam table on a clinical scanner. (d) Goat positioned in the head support frame. MR, magnetic resonance; MRgFUS, magnetic resonance guided focused ultrasound.

**FIGURE 2:**
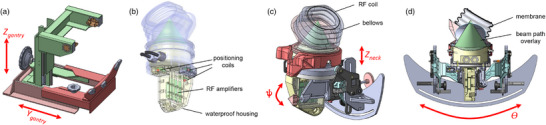
Design drawings of the three key components of the cervical spine MRgFUS system. Directions of motion are labeled with red arrows for each component: (a) positioning gantry, (b) transducer assembly and preamplifier housing, and (c), (d) the transducer assembly and acoustic coupling system in two different views with components individually labeled. All motion is achieved manually with visual gauges providing for accurate positioning. MRgFUS, magnetic resonance guided focused ultrasound.

The cervical spine MRgFUS system consists of three main components: (1) positioning gantry, (2) head support frame, and (3) transducer adjustment and coupling system. Figure 1 shows drawings in SolidWorks (SolidWorks Corp., Dassault Systèmes) and photos of the system installed on a 3T MRI with 70 cm wide bore (Vida, Siemens Healthineers, Erlangen, Germany). Figure 2 shows the individual components with labeled motion directions for each component. The positioning gantry was constructed of extruded fiberglass components and linear guide bearings that suspend the goat head and neck above the transducer system. Head position adjustments are available in the *Z* and *Y* directions, and transducer assembly adjustments are possible along the *Y* direction. Movement of the transducer assembly along Θ allows access from the left or right side of the neck for bilateral treatment with an angle of up to 30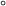 from vertical. Additional adjustments of the transducer in *Z* and ψ (tilt in *S*/*I* direction) allow for variable trajectories to the facet joint target.

The head support frame was specifically designed for goat anatomy for preclinical system validation. The frame is a clamping mechanism consisting of a bite bar on one side and a padded bar that fits behind the horns. The frame securely mounts to the positioning gantry with an optional chin tilt adjustment. The frame is attached to the goat during prep and allows the animal to be positioned in the supine position for posterior targeting.

The transducer assembly and acoustic coupling system (Figure 2b) consists of the phased array ultrasound transducer, three positioning coils^44^ for automatically determining transducer and focal spot position in the focused ultrasound control software environment (ThermoGuide, Image Guided Therapy, Pessac, France), supporting electronics for up to four MRI radiofrequency (RF) receive coils, and adjustment hardware for positioning of the transducer's focal spot. The transducer is secured within a water‐tight housing with an expandable 3M Tegaderm transparent film dressing (Solventum, St. Paul, Minnesota, USA; model number 1629, 20 cm × 30 cm) that is inflated with degassed water until it is acoustically coupled to the skin of the animal. A flexible rubber bellows holds a dedicated MRI RF receive coil against the animal around the coupling membrane. The system is designed to allow for bilateral treatment; in this preclinical configuration the bellows and coil are rotated 180 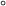 when the transducer assembly swings from one side to the other for bilateral access.

### 2.2 Ultrasound transducer

The ultrasound transducer in the cervical neck MRgFUS system is a 256‐element phased array transducer (Imasonic, Voray‐sur‐l'Ognon, France) that has been presented and evaluated in other applications.^45^ The transducer has a central frequency of 1 MHz, 256 transmit elements 5 mm in diameter positioned semi‐randomly over a section of a spherical surface with an 11 cm radius of curvature or focal length, and an additional 4 receive elements with more broadband characteristics that can be used to detect any cavitation activity. Radiation force balance measurements^46^ were obtained to calibrate the acoustic output of the transducer. Scanning hydrophone measurements were obtained with a calibrated hydrophone (HNA‐0400, Onda, Sunnyvale, CA) under free field conditions. Briefly, the hydrophone is mounted on a gantry with x and y stepper motors to allow a 2D scan to be acquired with 0.25 mm isotropic resolution over a 1.0 cm × 1.0 cm area center at the focal point of the ultrasound beam. The measured 2D complex pressure pattern is then propagated into a 3D volume matching the relevant portion of the simulated model size using the angular spectrum approach.^47^

### 2.3 Radiofrequency MR coils

Two types of RF MRI receive coils are integrated into the cervical neck MRgFUS system: transducer positioning coils and MR imaging coils.

Three positioning coils are placed circumferentially around the transducer assembly as seen in Figure 2b. These coils are used in conjunction with a simple MRI pulse sequence that has a 1D readout gradient applied in all three directions. The positions of the three coils form a triangle with unique side length, which allow the transducer position and focal spot location to be determined automatically in the ThermoGuide ultrasound control software environment. Details of the coil design, circuits, and localization technique are described in detail in Svedin, et al.^44^ The three coils are encased in waterproof housings to protect them from any leaking acoustic coupling fluid. The accuracy of the positioning coils was assessed by replacing the coupling bellows with a cone that contained a fiducial bead (benzonatate) fixed at the geometric focus of the transducer, allowing an accurate, manual assessment of the true position of the transducer's focal spot within the MR images.

Custom MR imaging coils were integrated into the cervical neck MRgFUS system. Two 10 cm diameter circular loop RF receive coils, made from RG‐316 coaxial cable, were designed to be positioned on either side directly on the surface of the subject's neck. One coil loop was designed to be an ultrasound shoot‐through coil loop and was placed around the rubber bellows, as shown in Figure 2b, allowing the ultrasound to pass through the coil. This also allowed the coil to be centered directly on the target volume of the subject. The second coil was placed on the subject's neck on the opposite side from the first loop. This second coil loop was used to improve the signal‐to‐noise ratio (SNR) in the center of the neck, near the FUS target volume, as compared to a single shoot‐through loop. Although the housing was designed to hold four RF amplifiers, we found that two coils provided sufficient SNR for this study and made coil setup during the imaging experiment easier to accomplish. After both coils were fixed into position on the subject's neck, the coils can be fine‐tuned and matched using remote tune/match circuits that were positioned near the preamplifiers at 12 cm from the coil loops.^48^ The remote tune and match circuits allow the coils to be tuned and matched to account for the variations in transducer/neck/water‐volume position that can occur as the transducer is positioned for different targets and target depths in a subject's neck. The remote tune and match circuits move the variable tuning components of the coil from the tightly spaced and complicated environment of the coils, bellows, support pads, and straps to a more accessible and convenient position.

Coil SNR evaluations were made using a 12 cm diameter by 20 cm length homogeneous copper sulfate phantom that was positioned in the cervical neck MRgFUS system. SNR was measured using a 2D gradient echo sequence (TR/TE = 500/5 ms, flip angle = 90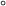, acquisition resolution = 2 mm ×2 mm × 3 mm, reconstruction resolution = 1 mm ×1 mm ×3 mm, FOV = 256 mm ×256 mm, BW = 260 Hz/pixel, single slice). SNR and noise correlation^44^ were calculated for two coil configurations: scanner body coil and the two dedicated loop coils placed around the bellows and on the opposite side of the subject's neck. SNR maps were generated over the cross‐sectional area of the phantom.

### 2.4 In vivo MRgFUS experiments

All animal experiments were approved by the University of Utah's Institutional Animal Care and Use Committee (protocol 20‐08008). Male goats (*N* = 5, Spanish/Boer cross breed, 10–12 months, 24.5–27.5 kg) were fasted a minimum of 12 h before the administration of anesthesia. Animals were first injected with midazolam (0.1–0.5 mg/kg) and ketamine (4–7 mg/kg) and then maintained with isoflurane (0.5%–3% with oxygen). Due to high ultrasound attenuation of goat skin, skin in the beam path was surgically removed before positioning the animal on the system. A stomach tube was placed to relieve gastric bloat.

All MRgFUS studies were performed in a 70 cm bore 3T scanner. After the headframe was attached to the animal, it was placed supine on the cervical spine MRgFUS system. Both the animal and positioning gantry could be moved to target the desired cervical level. After the transducer was fixed in position, the Tegaderm membrane was inflated with degassed water. MRgFUS targeting was done in the ThermoGuide control software. The transducer was located with the positioning coils, and the ultrasound beam path and geometric focus were overlaid on a T1‐weighted volumetric interpolated breath‐hold examination (VIBE) sequence with FOV = 192–256 mm in‐plane × 144–208 mm through‐plane, resolution = 0.7–2 mm in‐plane × 1–4 mm slice thickness, TR = 3.8–5 ms, TE = 1.44–2.3 ms, flip angle 6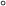, 1–4 averages, acquisition time = 56–165 s. Position of the animal and positioning gantry was manipulated in 1–2 iterations until accurate targeting was achieved, such that the desired focus was within 10 mm of the predicted geometric focus, well within the electronic steering range of the FUS transducer.

Both MR temperature imaging (MRTI) and MR acoustic radiation force imaging (MR‐ARFI)^49^ were performed with a 3D gradient recalled echo (GRE) segmented echo planar imaging (EPI) pulse sequence. MR‐ARFI was used to non‐invasively localize the focal spot in the near‐field in the muscle ∼5 mm proximal to the facet joint target. The MR‐ARFI pulse sequence used bi‐polar motion encoding gradients (MEG, 35 mT/m, 5 ms) in the slice encoding direction, inserted before the EPI readout. A short FUS pulse (3 ms, 120 W acoustic) was applied synchronously with the second MEG lobe. Two images with slice encoding perpendicular to the FUS propagation direction, with the FUS ON and OFF, were interleaved on the TR level as previously described^50^ to enable simultaneous MR‐ARFI and MR temperature imaging. Imaging parameters for combined MR‐ARFI/MRTI used FOV = 192 mm ×144 mm × 28.8 mm, acquisition resolution = 1 mm ×2 mm ×2.4 mm, reconstructed resolution = 0.5 mm × 0.5 mm × 1.2 mm, TR/TE = 22/14 ms, readout bandwidth = 1002 Hz/pixel, echo train length = 1, acquisition time = 76 s/MR‐ARFI map.

To correct for a potential offset between the selected target and actual focus, a sonication in the the muscle adjacent to the facet joint was performed, the spot location determined with MR‐ARFI, and the offset measured. The ultrasound focus was then electronically steered to the facet joint target, accounting for the MR‐ARFI measured offset, and MRgFUS sonications were performed and monitored via MRTI using the proton resonance frequency shift approach.^51^, ^52^ Imaging parameters for MRTI used FOV = 192 mm × 144 mm × 24 mm, acquisition resolution = 1 mm ×2 mm ×3 mm, reconstructed resolution = 1 mm ×1 mm ×1.5 mm, TR/TE = 42/13 ms, readout bandwidth = 1002 Hz/pixel, echo train length = 7, acquisition time = 3.7 s/3D volume, with fat‐saturation. For easier visualization of the heating during treatment, the MRTI volume was reformatted and temperature maps color‐overlaid in real‐time onto the previously acquired, axial VIBE images in ThermoGuide.

The precision of the MR temperature imaging was determined by calculating the standard deviation through time of voxels in a non‐heated area. Precision values were calculated for each sonication in regions of interest (ROI) defined in areas adjacent to the targeted focused ultrasound heating that did not experience heating.

After sonications at one vertebral level were completed, the Tegaderm membrane was slightly deflated and the transducer moved to the next target location. The membrane was then reinflated to ensure proper coupling before the procedure was repeated for the next vertebral level. We calculated the duration to mechanically move the transducer into the electronic targeting range of the next spine level, that is, the time between the end of the last sonication of the previous level and the beginning of the first MR‐ARFI sonication on the new level. This time included the deflation of the Tegaderm membrane, moving the transducer and imaging coils, inflating and coupling the membrane, transducer localization, and image acquisition of new target anatomy. The study goal was to treat three vertebral levels in each animal.

At the end of the treatment gadolinium (Gadoteridol, ProHance, Bracco Diagnostics Inc., Princeton, NJ, USA) was injected intravenously at a dose of 0.2 mmol/kg body weight and contrast enhanced imaging performed using a VIBE sequence with FOV = 184–256 mm in‐plane × 72–208 mm through‐plane, resolution = 1 mm in‐plane × 1–2 mm slice thickness, TR = 4.00–4.36 ms, TE = 1.44–1.95 ms, flip angle = 4
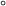
–6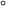, 1–4 averages, acquisition time = 165 –259 s. The goat was euthanized via transcardiac perfusion with cold 0.1 M phosphate buffer. The cervical spine and surrounding musculature were placed in 10% neutral buffered formalin (StatLab, McKinney, TX, USA). After 2 weeks, the samples were decalcified using Immunocal (StatLab, McKinney, TX, USA) and trimmed using a meat slicer to leave a thin (0.5 cm) slice containing the facet joint. Samples were processed and embedded in paraffin, sectioned at 5 μm, and stained with hematoxylin and eosin (H&E). The resulting H&E slide was imaged with an Axio Scan.Z1 Slide Scanner (Carl Zeiss Microscopy, Jena, Germany).

## 3 RESULTS

**FIGURE 3:**
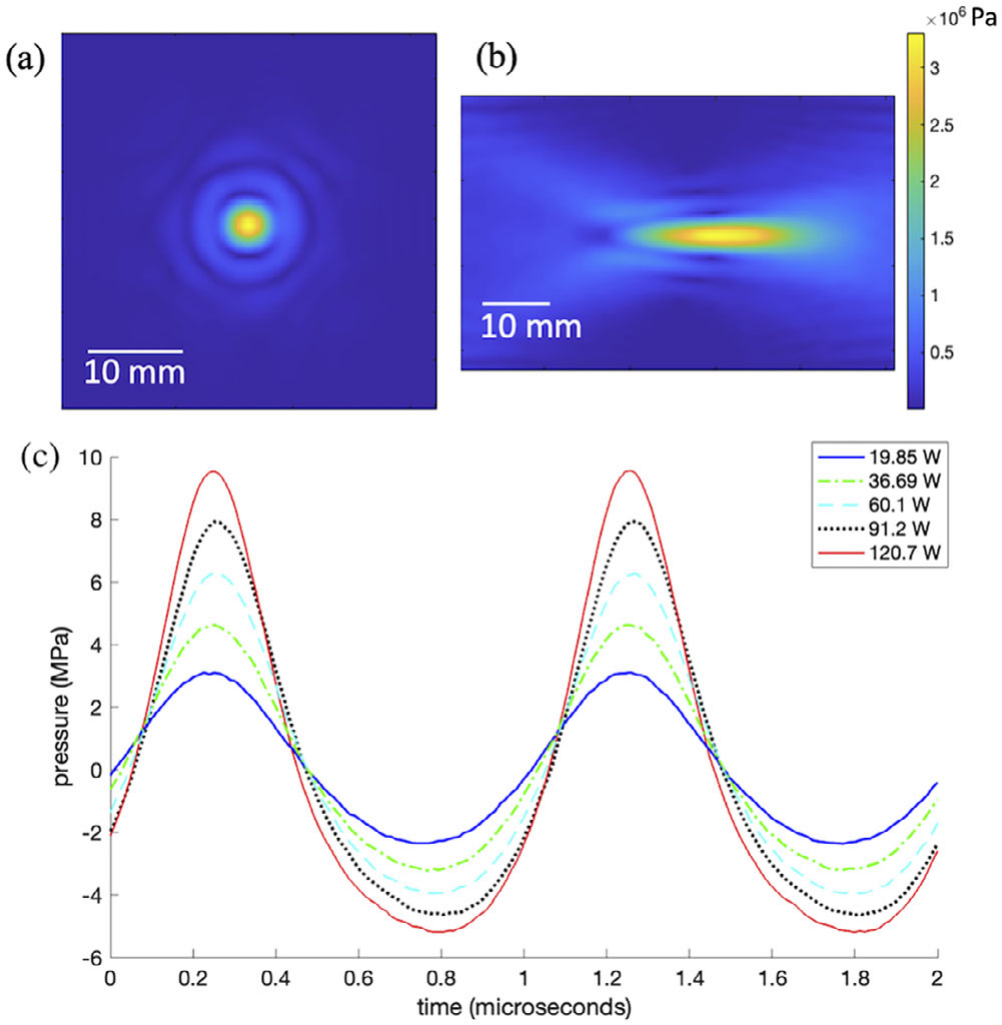
Performance characterization of the ultrasound transducer used in the presented cervical MRgFUS system. 2D hydrophone scan (Onda HNA‐0400) of the (a) transverse and (b) longitudinal patterns are shown, obtained at a transducer output of 20 acoustic W under free‐field conditions. In (b) the transducer is located to the left of the image. Ultrasound transducer pressure shown over a range of therapeutic power output levels: (c) The measured pressure wave over two acquired cycles. MRgFUS, magnetic resonance guided focused ultrasound.

Figure 3 shows the performance characterization of the ultrasound transducer that was used in this cervical MRgFUS system. Radiation force balance testing measured a mean acoustic efficiency of 53.3% over the full power operating range. Figure 3a,b shows 2D hydrophone scan planes of the ultrasound focal spot at a 20 acoustic Watt output under free‐field conditions. The longitudinal pressure pattern was obtained through propagating the transversal plane measurement using an angular spectrum technique.^53^ The full width half maximum size of the pressure profile was measured to be 2.0 mm ×2.0 mm ×12.1 mm. Figure 3c shows the pressure output of the ultrasound transducer over a range of therapeutic power output levels. The temporal pressure wave as well as the peak positive and peak negative pressure values are graphically shown.

**FIGURE 4:**
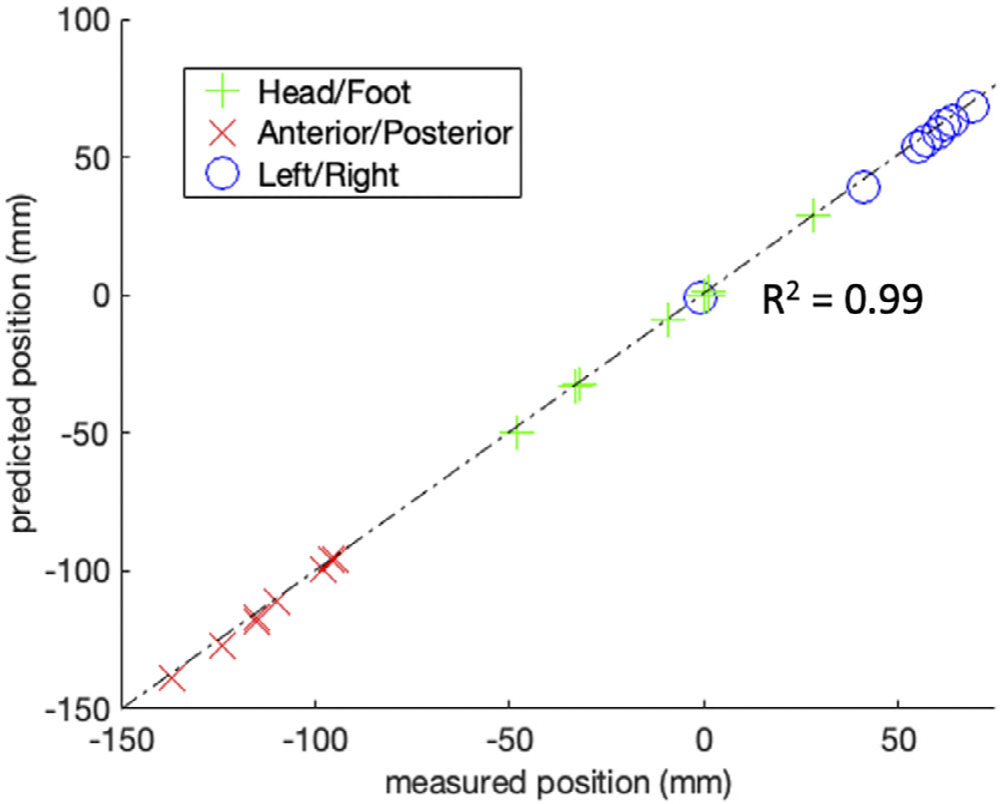
Comparison of the positioning coil prediction accuracy and measured focal point position of the transducer. The predicted and measured position is shown individually for all three MRI coordinate directions. The Euclidean distance error for all measured points was found to be 2.22±0.74 mm.

The performance of the positioning coils in accurately predicting the location of the geometric focus of the transducer is shown in Figure 4. The predicted and measured position of the geometric focus is assessed at eight points, with the dimensions of head/foot, anterior/posterior, and left/right plotted individually. The distance error for all measured locations was found to be 2.22 ± 0.74 mm across all orthogonal directions.

**FIGURE 5:**
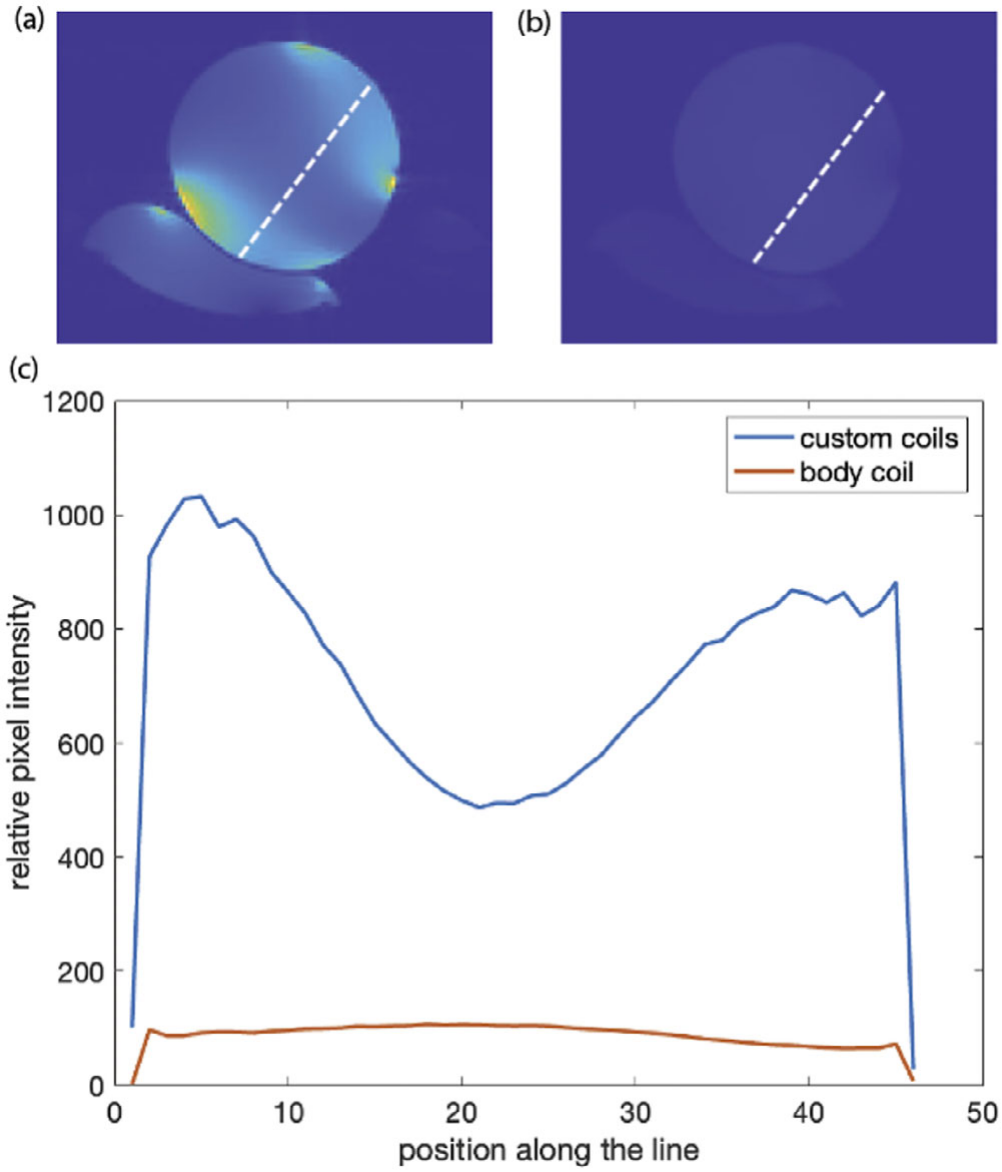
Image SNR of the cervical neck system using the dual coil system. The two coil conditions were evaluated with a cylindrical copper sulfate phantom. Axial images showing the dual coil (a) and body coil (b) SNR maps. Quantitative measurements (c) along the approximate ultrasound propagation direction (white dashed line). The dual coil condition provide an approximate 5 fold increase in SNR compared to the body coil in the center of the phantom. SNR, signal‐to‐noise ratio.

The image SNR for the dual 10 cm coil configuration compared to the scanner body coil is compared in Figure 5. The axial SNR view of the cervical neck system with coils on the copper sulfate phantom shows the qualitative spatial variation of SNR while a quantitative assessment is made across the approximate ultrasound propagation path, comparing the dual coil and body coil configurations. While the signal profile in the dual coil configuration is inhomogeneous, with higher SNR closer to the coils, the SNR in the middle of the phantom is still 5‐fold higher than compared to the body coil only condition. This coil design provided the required SNR for both visualization of the targeting region for treatment planning and assessment and for the achievement of adequate temperature precision measurements from the MR temperature imaging in the complicated environment of the cervical spine. Coil tune was confirmed at the beginning of the animal procedure; the time required for fine tuning and matching depends on the accuracy of the initial guess but took less than 5 min and did not need to be repeated when moving the transducer assembly to a different spinal level. Because neck size was very similar between animals, tune, and match adjustments were not necessary after the first in vivo goat experiment.

**FIGURE 6:**
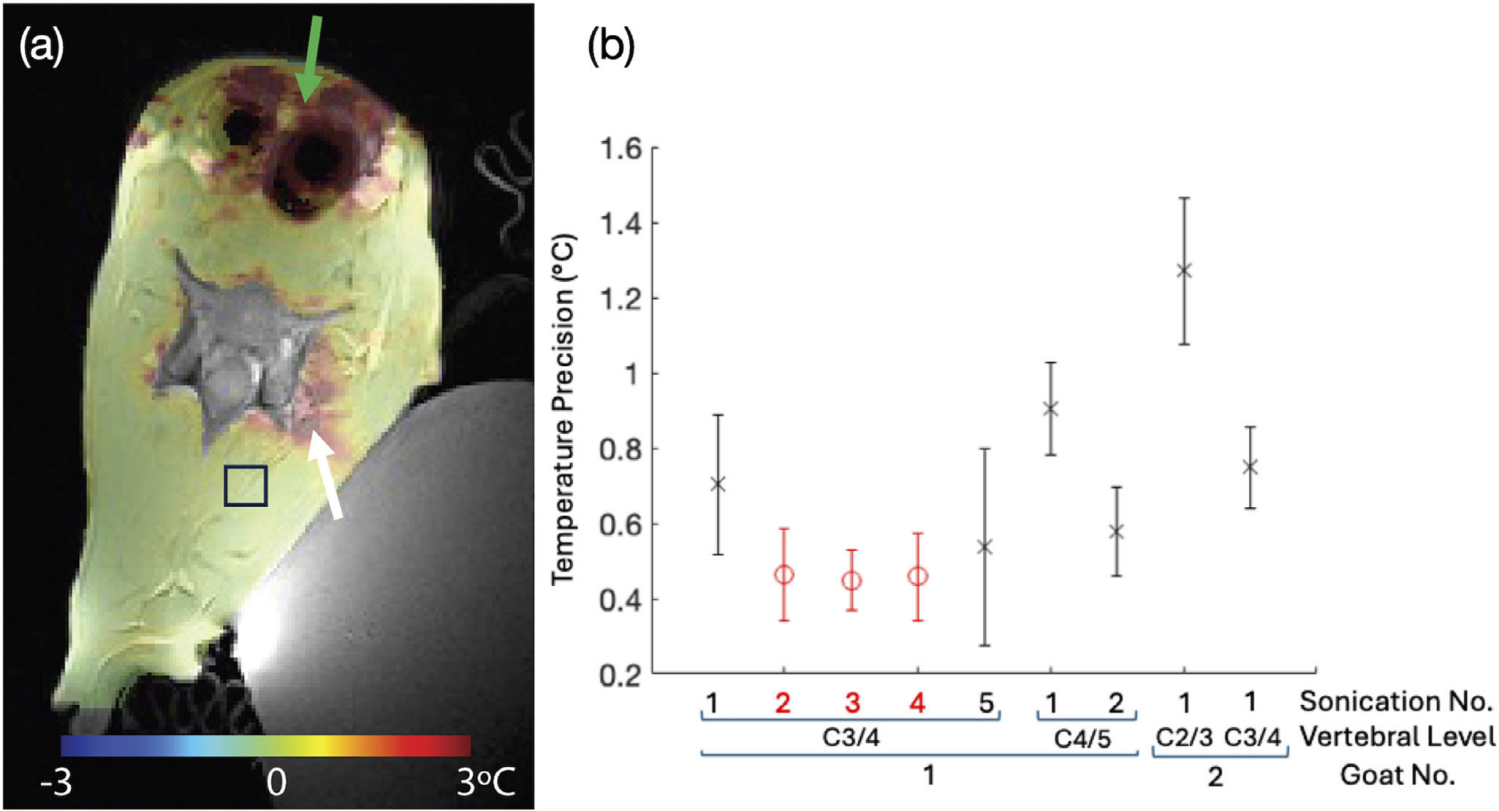
The MRTI precision during one sonication at level C3‐4 in one goat overlaid onto the reformatted anatomical slice with a mask applied to bone, air, and coupling balloon (a). The precision in the defined square ROI (25×25 voxels) is 0.45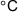. The area indicated by the white arrow is where the heating occurred during the sonication. The area at the top of the image indicated by the green arrow has a decreased measurement precision due to the motion and pulsation/flow artifacts of the esophagus, trachea and large veins and arteries in the region. Spatial mean and standard deviation over the precision values in the ROIs for sonications at different vertebral levels in two goats (b). Values in red show sonications where no motion was observed over the time course of the sonication. MRTI, MR‐temperature imaging; ROI, regions of interest.

Figure 6a shows the magnetic resonance temperature imaging precision in one goat. The MRTI data were acquired axially and the image shows one slice overlaid on an axial high resolution T1‐weighted image. A mask was applied to low signal areas by thresholding the image magnitude of the underlying MRTI image, masking out bone, air, and the coupling balloon. The precision in the non‐heated region‐of‐interest was 0.45 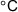 for the shown sonication. Over the seven sonications in this animal, performed over three cervical levels, the mean temperature measurement precision was 0.58 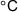. Figure 6b shows the individual precision values (spatial mean and standard deviation in the ROIs) for different sonications at the different vertebral levels in two goats. Most sonications, except the ones shown in red in the graph, had small amounts of motion as observed by watching the magnitude images over time. Areas of reduced precision were seen around sources of motion artifacts, including the esophagus (with rumen tube), trachea (with breathing tube), and large blood vessels with pulsatile blood flow (green arrow). In addition, temperature precision is decreased right next to the vertebral bone, which is due to partial volume effect in bone containing voxels as the underlying MRTI image is acquired with 1 mm ×2 mm ×3 mm resolution. Those partial‐volume voxels have lower signal and therefore lower precision than the surrounding soft tissue, but not low enough to be masked out by the thresholding operation.

**FIGURE 7:**
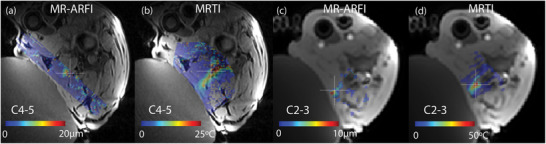
Demonstration of MR‐ARFI and MRTI utilized for MRgFUS in the cervical spine in two different goats with color overlays reformatted onto the identical anatomical slice in each goat. The MR‐ARFI displacement was measured at a location in the muscle, proximal to the intended target to verify the focus location. The MRTI was measured at the peak temperature during a focused ultrasound sonication and shows ablation at the targeted facet joint. The crosshair overlay shows the location of the predicted focus in muscle before correction (a,c) and the predicted focus after correction (c,d) at the facet joint in the targeted slices for MR‐ARFI and MRTI, respectively, which are targeting different locations. MR‐ARFI, MR‐acoustic radiation force imaging; MRTI, MR‐temperature imaging; MRgFUS, magnetic resonance guided focused ultrasound.

Figure 7 demonstrates how the MRgFUS cervical neck system uses MR‐ARFI to measure a potential offset between the predicted focus (determined through MR positioning coils locating the transducer) and actual sonication focus, and how the subsequent ablation of the facet joint target is monitored with MRTI at different cervical levels in the goat. Color maps of the reformatted imaging volumes showing MR‐ARFI displacement and MRTI temperature rise are overlaid onto the anatomical images acquired at each vertebral level. After the transducer is positioned such that the focus is located well within the electronic steering range, a spot is selected in muscle adjacent to the facet joint capsule and an MR‐ARFI sonication is performed. Quantitative displacement images show the location of the actual focus compared to the predicted focus (white crosshair) and corrections can be applied. The temperature rise measured in this combined MR‐ARFI/MRTI acquisition was negligible showing that MR‐ARFI allows focus localization without heat deposition. In Figure 7a, the MR‐ARFI slab was not centered on the sonication focus (white crosshair), so it is not clear if there was additional displacement outside the imaged area. Figure 7c shows the maximum displacement slightly toward the transducer, but within the expected targeting precision shown in Figure 4. After the offset between predicted and actual focus was quantified, the focus was electronically steered to the facet joint capsule and a correction to the focus offset was applied for subsequent sonications. Figure 7b,d shows the temperature rise at the target after correction. Figure 7d shows a very good match between the predicted focus and the hottest area. From the MRTI image in Figure 7b, it is difficult to identify the actual focus due to the heterogeneous tissues and tissue interfaces (bone, muscle, fascia). Therefore, due to the high ultrasound absorption in bony targets, the hottest temperature reached is not necessarily at the focus and preferential heating around tissue interfaces can occur.

**FIGURE 8:**
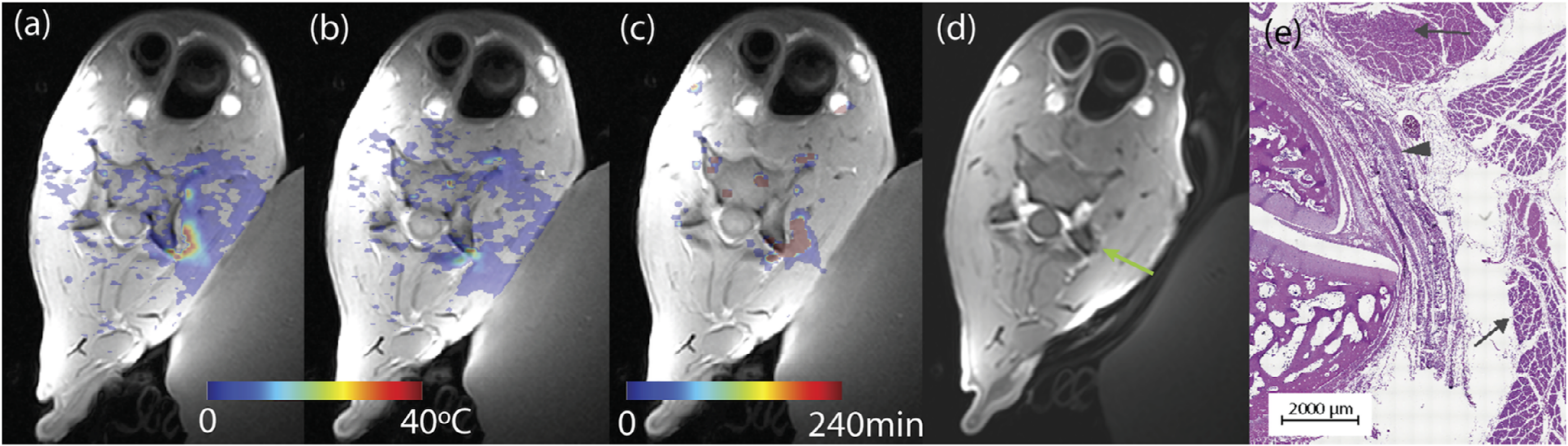
Example of MRgFUS heating at a C2‐3 level. (a) and (b) show the peak temperature for two of three individual sonications performed and (c) the cumulative thermal dose for all sonications indicates successful ablation. (d) T1‐weighted contrast‐enhanced image at that treated level after the ablation shows perifacet enhancement indicating increased contrast accumulation due to increased blood flow at the ablation site (green arrow). (e) H&E histology of the facet joint and adjacent tissue. Arrows: areas of thermal damage in muscle tissue; arrowhead: spinal nerve at site of ablation. MRgFUS, magnetic resonance guided focused ultrasound.

Figure 8 shows the ablative treatment and outcome at the C2‐3 level in one goat. A single axial slice of the 3D thermometry at the time of peak heating for two (a, b) of three MRgFUS ablations performed at this level, as well as a cumulative thermal dose map (c) show the ability of the MRTI to measure precise temperatures around the region of interest. A contrast‐enhanced T1‐weighted image obtained within 15 min after the conclusion of ablation shows areas of edema and necrosis around the facet joint (d). H&E histology of the excised tissue at this level demonstrates the presence of thermal damage in the targeted area and closely aligns with the MR imaging findings.

In the five animals in this study, successful treatments were achieved at three vertebral levels in three animals. In one animal, two joints were targeted and successfully treated; the third level was not attempted as a building wide power outage delayed the start of the experiment. In one animal, only one joint was targeted but not successfully treated due to low image quality from a coil coupling issue between the two loop coils and a software communication issue between MR scanner and ThermoGuide control software, which caused the control software to crash during sonication. In the three animals with successful treatments on three levels, the time to move the system from one level to the next was measured to be 24.5 min on average, ranging from 18–32 min.

## 4 DISCUSSION

The cervical‐spine specific MRgFUS system presented here was designed for bilateral treatments in humans but includes modifications to the goat anatomy to allow for preclinical evaluation. The goat model was chosen because neck diameter, vertebral body size and shape, and surrounding tissues are similar to human anatomy. The bone mineral density in goat vertebrae (0.39–0.42 g/${\mathrm{cm}}^{3}$) is considerably higher than in humans (0.12–0.14 g/${\mathrm{cm}}^{3}$).^54^ For this feasibility study measuring the accuracy of targeting, the bone density difference is not relevant, but may need to be considered if the goat model is used for the prediction of energy transmission and absorption within the bone. The positioning gantry with head support frame was specifically built to support the goat head and neck. The transducer positioning assembly and coupling system have small modifications that can be easily translated back into a clinical prototype. Although the system was designed for bilateral treatments, we used unilateral treatments in our in vivo goat experiments using the collateral side for comparison. In a bilateral treatment, the transducer assembly would be rotated from one side of the neck to the other. In the goat system, we added a slanted adapter (seen in Figure 2b–d) between the transducer housing and the bellows to better conform to the narrow and pointed goat neck, which also would need to be rotated by 180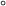 between treatment sides. Since the human neck is much rounder compared to the goat, the bellows are directly mounted onto the transducer housing such that rotating the transducer to the other side does not require this additional step.

MR temperature measurement precision with the two receive coils positioned around the neck was below 1 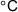 in all vertebral levels in the area of treatment (Figure 6) which is adequate for monitoring ablation treatments where the goal is to reach a temperature of 60 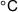 (or a 23 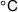 temperature rise from baseline). Temperature precision was reduced considerably near the visceral and carotid spaces; however, this area is well outside the treatment path and of no physiological relevance. Future precision studies in humans will determine if the MRTI sequences employed here are sufficient or if additional motion compensation techniques are necessary.

Accuracy measurements showed a 2.22 ± 0.74 mm distance error between the actual geometric focus of the transducer and the focus position predicted by the three positioning coils (Figure 4). This error is well within the electronic steering range of the transducer, which is about 5 cm transverse to and 10 cm along the the US propagation direction. Once the transducer is in treatment position for targeting a certain vertebral level, the focus location can be further confirmed or adjusted with MR‐ARFI.

Clinical MRgFUS treatments such as essential tremor, perform one or several verification sonications at sub‐lethal energy levels to confirm the location of the target with MRTI, before increasing the energy to create permanent lesions. This focal spot verification also takes potential beam aberration caused by the intervening tissue structure into account. Similarly, MR‐ARFI is being investigated as a non‐thermal method to determine the position of the ultrasound focus and calibrate the intensity at the target by measuring the tissue displacement, which is of particular interest in transcranial neuromodulation applications in the brain. These methods work well in soft tissues, but are more complicated when targeting bony structures as the ultrasound gets absorbed and reflected at the bone soft tissue interface. In addition, for bone applications, such as lumbar facet joints and painful bone metastases, the ultrasound focus may be placed behind the surface of the bone on purpose to create a larger ablation area at the highly absorbing bone surface, which make it difficult to pinpoint the exact focus based on MR imaging. For this reason, feedback from MRTI and MR‐ARFI cannot be used to verify that the transducer is focused on the desired facet joint target. For verification in this scenario, we chose to perform MR‐ARFI in the muscle adjacent to the facet joint target by electronically steering toward the transducer along the US propagation direction. Since only a small amount of steering is necessary to move the focus back to the target, tissue changes along the beam path should be minor between verification and target position. Although we used MR‐ARFI for localizing the focus and corrected for the offset in subsequent ablations at the facet joint target, we did not verify the correction with an additional MR‐ARFI sonication in this study. Therefore, for translation of the MR‐ARFI localization method into the clinic, further validation of the accuracy of MR‐ARFI in muscle tissue is necessary as the tissue displacement may depend on the direction of the muscle fibers with respect to the direction of the beam. In addition, a comparison of MR‐ARFI with low‐temperature sonications for focal spot confirmation is needed to determine which method is more suitable for this application.

Due to the high ultrasound attenuation of goat skin, we had to surgically remove the skin in the beam path. Without skin removal, skin burns occurred that further increased attenuation at the skin interface and therapeutic temperatures at the target could not be reached. This problem appears to be specific to goat skin and has not occurred in similar studies by our group in the lumbar spine in swine which also used a 1 MHz transducer. Similarly, no skin burns were reported with the 1 MHz transducer used in the Neurolyzer XR clinical trial.^41^ Comparing the ultrasound attenuation in radiation force measurements at 1 MHz showed higher attenuation in goat skin (0.396 Neper/cm ± 0.026 (standard error; measured in three goats) compared to pig skin (0.10 ± 0.013 (standard deviation); measured in one pig).^55^ The goat skin attenuation is also higher compared to reported literature values for human skin (0.21 Neper/cm).^56^ Therefore, despite the favorable neck and cervical spine anatomy, the goat model may not be the best preclinical model for evaluating focused ultrasound devices.

### 4.1 Limitations

Limitations include the small number of animals in the study and unilateral treatments only, which do not demonstrate how the system performs when moved from one side to the other. In addition, goat neck anatomy and goat skin ultrasound attenuation differ from those of humans, which required skin removal to achieve ablative temperatures at the facet joint target. Finally, further evaluation of MR‐ARFI in muscle tissue for focal spot confirmation and comparison to MRTI in a sublethal sonication is needed.

## 5 CONCLUSION

We presented the design and preclinical evaluation of a neck‐specific MR‐guided focused ultrasound device for ablative treatment of facet joints in the cervical spine. The device design allows for bilateral treatment of vertebral levels from C2‐3 to C6‐7. Integrated MR‐coils allow for accurate positioning of the transducer and provide sufficient SNR to guide and monitor the procedure. MR‐acoustic radiation force imaging and MR‐temperature imaging provide focal spot localization and allow treatment monitoring of the procedure. The clinical translation of this design will potentially lead to a non‐invasive treatment for facet joint related neck pain.

## CONFLICT OF INTEREST STATEMENT

The authors Payne, Hadley, Merrill, Shah, and Rieke have a potential research conflict of interest due to intellectual property. The authors Payne, Shah, and Rieke are founders of Sonovis Therapy Inc., related to the technology described in the manuscript. A management plan has been created to preserve objectivity in research in accordance with University of Utah policy.

## Data Availability

Imaging data will be shared upon request by the corresponding author.
